# Epidemiology of Congenital Rubella Syndrome (CRS) in India, 2016-18, based on data from sentinel surveillance

**DOI:** 10.1371/journal.pntd.0007982

**Published:** 2020-02-03

**Authors:** Manoj Murhekar, Sanjay Verma, Kuldeep Singh, Ashish Bavdekar, Naveen Benakappa, Sridhar Santhanam, Gajanan Sapkal, Rajlakshmi Viswanathan, Mini P. Singh, Vijaya Lakshmi Nag, Sadanand Naik, Munivenkatappa Ashok, Asha Mary Abraham, Devika Shanmugasundaram, R. Sabarinathan, Valsan Philip Verghese, Suji George, Ravinder Kaur Sachdeva, Jyoti Kolekar, S. Manasa, Jagat Ram, Madhu Gupta, Manoj K. Rohit, Praveen Kumar, Parul Chawla Gupta, R. K. Ratho, Sanjay Kumar Munjal, Urvashi Nehra, Daisy Khera, Neeraj Gupta, Nidhi Kaushal, Pratibha Singh, Ravisekhar Gadepalli, Neelam Vaid, Sandeep Kadam, Sanjay Shah, S. Mahantesh, Vykuntaraju K. Gowda, Pradeep Haldar, M. K. Aggarwal, Nivedita Gupta

**Affiliations:** 1 ICMR–National Institute of Epidemiology, Chennai, India; 2 Postgraduate Institute of Medical Education and Research, Chandigarh, India; 3 All India Institute of Medical Sciences, Jodhpur, India; 4 KEM Hospital, Pune, India; 5 Indira Gandhi Institute of Child Health, Bengaluru, India; 6 Christian Medical College and Hospital, Vellore, India; 7 ICMR–National Institute of Virology, Pune, India; 8 ICMR–National Institute of Virology, Bangalore Unit, Bengaluru, India; 9 Ministry of Health and Family Welfare, Govt of India, New Delhi, India; 10 Indian Council of Medical Research, New Delhi; University of Queensland, AUSTRALIA

## Abstract

**Background:**

Government of India is committed to eliminate measles and control rubella/congenital rubella syndrome (CRS) by 2020. In 2016, CRS surveillance was established in five sentinel sites. We analyzed surveillance data to describe the epidemiology of CRS in India.

**Methodology/Principal findings:**

We used case definitions adapted from the WHO-recommended standards for CRS surveillance. Suspected patients underwent complete clinical examination including cardiovascular system, ophthalmic examination and assessment for hearing impairment. Sera were tested for presence of IgM and IgG antibodies against rubella. Of the 645 suspected CRS patients enrolled during two years, 137 (21.2%) were classified as laboratory confirmed CRS and 8 (1.2%) as congenital rubella infection. The median age of laboratory confirmed CRS infants was 3 months. Common clinical features among laboratory confirmed CRS patients included structural heart defects in 108 (78.8%), one or more eye signs (cataract, glaucoma, pigmentary retinopathy) in 82 (59.9%) and hearing impairment in 51. (38.6%) Thirty-three (24.1%) laboratory confirmed CRS patients died over a period of 2 years. Surveillance met the quality indicators in terms of adequacy of investigation, adequacy of sample collection for serological diagnosis as well as virological confirmation.

**Conclusions/Significance:**

About one fifth suspected CRS patients were laboratory confirmed, indicating significance of rubella as a persistent public health problem in India. Continued surveillance will generate data to monitor the progress made by the rubella control program in the country.

## Author summary

Rubella infection during the first trimester of pregnancy can affect the fetus, resulting in spontaneous abortion, stillbirth or an infant born with a combination of birth defects known as congenital rubella syndrome (CRS). Government of India is committed to eliminate measles and control rubella/CRS and has completed nationwide immunization campaigns using measles-rubella vaccine targeting children aged 9 months to 14 years. A case-based surveillance for CRS is one of the strategies for achieving elimination. The Indian Council of Medical Research and the Ministry of Health and Family Welfare initiated surveillance for CRS in five sentinel sites to estimate the disease burden. During 2016–18, the surveillance sites enrolled 645 suspected CRS patients, 137 (21.2%) were classified as laboratory confirmed CRS and 8 (1.2%) as congenital rubella infection. Common clinical features among laboratory confirmed CRS patients included structural heart defects (78.8%), one or more eye signs (cataract, glaucoma, pigmentary retinopathy (59.9%)) and hearing impairment (38.6%). Thirty-three (24.1%) laboratory confirmed CRS patients died over a period of 2 years. The surveillance data indicated significance of rubella as persistent public health problem in India.

## Introduction

In India, rubella is a common cause of febrile illness with rash among children. Postnatally acquired rubella infections are mild in nature and are seldom associated with complications [1]. However, the public health importance of rubella is due to teratogenic effects of rubella infection occurring in pregnant women [1]. Infections just before conception or during the first trimester of pregnancy can severely affect fetus, resulting in spontaneous abortion, stillbirth or an infant born with a combination of birth defects known as congenital rubella syndrome (CRS) [1,2]. In countries where rubella infection is endemic, CRS is an important cause of severe birth defects [3]. It has been estimated that during 1996–2010, globally 105,000 infants with CRS were born every year, 38% of which were from India [4].

Rubella vaccine is safe and highly effective [3]. More than 95% of recipients older than 11 months seroconvert with one dose of vaccine and antibody responses are detectable for a long period [3,5]. With successful vaccination programs, several countries have eliminated rubella or have substantially reduced the burden of CRS [6]. In 2013, during the 66^th^ session of the Regional Committee of the World Health Organization South-East Asia Region (SEAR), 11 SEAR countries adopted goals to eliminate measles and control rubella and congenital rubella syndrome by 2020 [6]. In India, a phased nationwide supplementary immunization activity using measles-rubella vaccine targeting children aged 9 months to 14 years commenced in 2017 [7] and has been completed in most Indian States. The Indian Council of Medical Research and the Ministry of Health and Family Welfare, Government of India, initiated surveillance for CRS in five sentinel sites in five Indian States in November 2016 with the objective of estimating disease burden [8]. In long term, this surveillance network will generate data to monitor the progress made by the rubella control program. This report describes the epidemiology of CRS in India, based on two years of sentinel surveillance.

## Methods

### Sentinel sites

All sentinel sites were tertiary care hospitals, located in urban areas. These facilities cater to a large population, not only within the city where they were located, but also neighboring districts within the State as well as neighboring States. Additional details of the surveillance sites are provided in supplementary table (S1 Table).

### Case finding and data collection

CRS surveillance is focused on identifying suspected CRS patients (a) during clinical examination of babies born at sentinel sites or (b) infants aged 0–11 months attending pediatrics, otorhinolaryngology, ophthalmology, and cardiology outpatient departments (OPDs) of the sentinel hospitals. We used case definitions adapted from WHO-recommended standards for CRS surveillance [8,9]. For the purpose of case finding, a case of suspected CRS was defined as an infant meeting any one of the following five criteria: (a) structural heart defects confirmed by echocardiography (b) hearing impairment confirmed by brainstem evoked response audiometry (BERA), or auditory steady-state response (ASSR) or two ‘refer’ otoacoustic emission (OAE) tests, (c) one or more of the following eye signs: cataract, microphthalmos, microcornea, congenital glaucoma, and pigmentary retinopathy (d) maternal history of suspected or confirmed rubella infection during pregnancy or (e) strong clinical suspicion.

At each sentinel site, the surveillance team consisted of a pediatrician (surveillance coordinator), cardiologist, ophthalmologist, ENT surgeon, obstetrician and microbiologist. Suspected CRS patients were identified during newborn examination or from the patients presenting in out-patient departments of clinical specialties. These patients were referred to the surveillance coordinator for detailed clinical examination. Patients were enrolled in the surveillance after obtaining written informed consent of the parents and information about demographic, epidemiologic, and clinical details were recorded in standardized case-report form. Patients were further referred to undergo eye, hearing and cardiac evaluation by respective departments. Suspected heart defects were confirmed by echocardiography. If hearing impairment was clinically suspected, BERA or ASSR audiometry was done as a diagnostic test.

### Laboratory investigations

#### Specimen collection

One ml of blood was collected from suspected CRS patients in plain blood collection tube. Serum was separated and stored at -20°C. Additional blood sample was collected after four weeks of collection of first specimen from (a) infants aged <1 month at the time of first blood collection and whose first blood sample was negative for IgM antibodies against rubella, as IgM seropositivity can be delayed until after the first month of life (b) infants aged 6–11 months whose first blood sample was negative for IgM antibodies against rubella but positive for IgG antibodies and (c) infants with indeterminate IgM or IgG antibody result on testing first blood sample. In the first year of surveillance, oro-pharyngeal (OP) swabs were collected from infants aged ≤ 5 months; thereafter, OP swabs were collected from all suspected CRS infants irrespective of the age. The data were entered in the web-based data entry portal.

#### Serology

The laboratory diagnosis of CRS was based on detection of IgM antibodies against rubella, or demonstrating a sustained level of IgG antibodies, as determined on at least two specimens collected at least 1 month apart [10]. At surveillance sites, sera from all suspected CRS patients were tested for presence of IgM rubella antibodies using commercial ELISA kit (Euroimmun, Luebeck, Germany). Sera from infants aged between 6–11 months negative for IgM antibodies were also tested for IgG rubella antibodies (Euroimmun, Luebeck, Germany).

#### RT-PCR

OP swabs from all the sites were transported to the ICMR-National Institute of Virology, Pune. These samples were tested for presence of rubella virus RNA by reverse-transcriptase polymerase chain reaction (RT-PCR) and representative samples positive for RT-PCR were genotyped as per WHO guidelines [10]. QIAamp Viral RNA Mini Kit (Qiagen, Hilden, Germany) was used for viral RNA extraction and Qiagen one step RT-PCR kit (Qiagen, Hilden, Germany) was used for genotyping.

#### Monitoring for viral excretion

The follow-up of laboratory confirmed CRS patients for monitoring of viral excretion was initiated in the second year of surveillance. For this, follow-up OP swabs were collected periodically and tested with RT-PCR.

### Final case classification

Final case classification was based on the presence of group A or group B clinical signs as well as laboratory results. The signs in group A included cataract, congenital glaucoma, pigmentary retinopathy, congenital heart defect, or hearing impairment. The signs in group B include microcephaly, developmental delay, meningo-encephalitis, splenomegaly, purpura, radiolucent bone disease, or jaundice with onset within 24 hours after birth. Based on the clinical signs and laboratory results, suspected CRS patients were classified into one of the following [9]:

- Laboratory-confirmed CRS: Infant having at least one sign from group A and meeting one of the following laboratory criteria: detection of rubella IgM antibody; or sustained level of rubella IgG antibodies, as determined on at least two occasions at age 6–12 months, in the absence of receipt of rubella vaccine.
- Clinically compatible CRS: Infant who has two clinical signs from group A or one from group A and one from group B, but from whom adequate specimen could not be collected.
- Congenital rubella infection: Infant who meets the laboratory criteria for CRS, but does not have any sign from group A.
- Discarded case: A suspected CRS case with adequate specimen, not meeting the laboratory-confirmed case definition, or a suspected case without an adequate laboratory specimen and not meeting the case definition of clinically compatible CRS.

### Data analysis

#### Descriptive epidemiology

The data were analyzed to describe the distribution of laboratory confirmed CRS patients by place (district of residence) and person (age, sex, age of mother, clinical details) characteristics.

#### Surveillance performance indicators

We calculated WHO recommended indicators pertaining to adequacy of investigation, adequacy of specimen collection and testing, adequacy of specimens for viral detection, monitoring for viral shedding, timeliness of case detection, timeliness of specimen transport and timeliness of reporting laboratory results [11, 12]. Adequacy of investigation was defined in terms of (a) collection of following data: demographic details (name, place of residence, sex, date of birth), date of reporting; date of investigation, date of specimen collection, clinical outcome and age of mother and (b) completeness of clinical evaluation for heart defects, eye signs and hearing.

### Human participants protection

The Institutional Ethics Committees of ICMR-National Institute of Epidemiology, Chennai and of all surveillance sites approved the surveillance protocol. Written informed consent was obtained from the parents, prior to enrolling suspected CRS patients in surveillance.

## Results

### Suspected CRS

Surveillance sites enrolled 645 suspected CRS infants from November 2016 -December 2018. Their mean age was 3.7 months [standard deviation (SD): 3.4] and 377 (58.5%) were boys. One hundred and twenty five (19.4%) cases were identified during newborn examination, while the first contact point of the remaining infants was pediatrics (n = 348, 54.0%), ophthalmology (n = 111, 17.2%), cardiology (n = 47, 7.3%) and otorhinolaryngology (n = 14, 2.2%) OPDs.

Two-thirds of the suspected CRS patients were enrolled based on structural heart defect (n = 428, 66.4%), 229 (35.5%) had one or more eye signs while 81 (12.6%) had hearing impairment. Mothers of 100 (15.5%) infants had a history of febrile illness during their pregnancy (Table 1). Overall, 471 (73%) infants had one, 127 (19.7%) had two and 47 (7.3%) had >2 criteria of suspected CRS.

**Table 1:** Characteristics of suspected cases of congenital rubella syndrome (CRS), Congenital Rubella Surveillance System, India, November 2016–December 2018 (N = 645).

Characteristic of patients	Number	%
Criteria for suspecting CRS*		
Structural heart defect	428	66.4
Eye signs	229	35.5
Maternal history of fever with rash during pregnancy	100	15.5
Hearing impairment	81	12.6
Clinically suspected	30	4.7
**Age at diagnosis**		
<1 month	146	22.6
1–5 months	304	47.1
6–11 months	195	30.2
**Sex**		
Male	377	58.5
Female	268	41.6
**Age of mother**		
17–25	347	53.8
26–30	218	33.8
31–35	64	9.9
>35	13	2.0
Not available	3	0.5

(*Patients had >1 criteria of suspected CRS)

Of the 645 suspected CRS infants, 137 (21.2%) were classified as laboratory confirmed CRS, 8 (1.2%) as congenital rubella infection, 38 (5.9%) as clinically compatible cases, while the remaining 462 (71.6%) cases were classified as discarded cases. The laboratory confirmed CRS infants were from 14 Indian States (S1 Fig). The mean age of mothers of laboratory CRS patients was 25.8 years (SD: 3.7).

Mothers of 114 suspected CRS infants, including 26 laboratory confirmed CRS infants, had delivered in four sentinel surveillance health facilities. Considering the annual number of deliveries in these facilities as the denominator, the minimum annual reporting rate for suspected and laboratory confirmed CRS in these sentinel sites ranged from 6.5 and 1.2 per 10,000 live births at Christian Medical College, Vellore; 7.6 and 1.3 per 10,000 at Postgraduate Institute of Medical Education and Research, Chandigarh; 25.7 and 5.8 per 10,000 at KEM Hospital, Pune and 68.0 and 5.7 per 10,000 at All India Institute of Medical Sciences, Jodhpur.

### Clinical details

Table 2 describes clinical findings among suspected and lab-confirmed CRS patients. Among 137 laboratory confirmed CRS infants, 108 (78.8%) had structural heart defects, 82 (59.9%) had eye signs from group A (8 patients had more than 1 eye sign from group A) while 51 (38.6%) had confirmed hearing impairment (Table 2). Heart defects were further classified as single (n = 69, 63.9%) and complex heart defects (n = 39, 36.1%). Patent ductus arteriosus (n = 44, 63.8%), ventricular septal defect (n = 12, 17.4%) and atrial septal defect (n = 12, 17.4%) were the commonest single heart defects (S2 Table). Congenital cataract (n = 68, 49.6%), pigmentary retinopathy (n = 13, 9.5%) and congenital glaucoma (n = 9, 6.6%) were the commonest group A ophthalmic signs. Group B signs among the laboratory confirmed patients included microcephaly (n = 78, 56.9%), developmental delay (n = 48, 35.0%), splenomegaly (n = 29, 21.2%) and purpura (n = 8, 5.8%). Of 137 laboratory confirmed patients, 33 died over a period of 2 years, with a case fatality of 24.1%. The exact cause of death among these children could not be determined, as most died at home or within few hours after admission.

**Table 2:** Clinical characteristics of suspected and laboratory confirmed CRS cases.

Clinical details	Suspected CRS patients		
	All (n = 645) (%)	Laboratory confirmed CRS (n = 137) (%)	Discarded cases (n = 462) (%)
**Hearing assessment**			
Hearing impairment^*^	110/609 (18.1)	55/136 (40.4)	48/433 (11.1)
Confirmed hearing impairment^**^	92/591 (15.6)	51/132 (38.6)	35/420 (8.3)
Mild	11 (12.0)	5 (9.8)	4 (11.4)
Moderate	19 (20.6)	8 (15.7)	11 (31.4)
Severe to profound	62 (67.4)	38 (74.5)	20 (57.1)
**Ophthalmic examination**			
Cataract	179 (27.8)	68 (49.6)	100 (21.7)
Congenital glaucoma	25 (3.9)	9 (6.6)	15 (3.3)
Microphthalmos	42 (6.5)	17 (12.4)	22 (4.8)
Pigmentary retinopathy	23 (3.6)	13 (9.5)	9 (2.0)
Microcornea	26 (4.0)	9 (6.6)	15 (3.3)
**Cardiac examination**			
Structural heart defect	440/641 (68.6)	108/137 (78.8)	300/459 (65.4)
**General examination**			
Splenomegaly	87 (13.5)	29 (21.2)	46 (10.0)
Hepatomegaly	196 (30.4)	44 (32.1)	128 (27.7)
Purpura	15 (2.3)	8 (5.8)	5 (1.1)
Jaundice	100 (15.5)	16 (11.7)	73 (15.8)
Rash	43 (6.7)	18 (13.1)	19 (4.1)
Lymphadenopathy	11 (1.7)	1 (0.7)	8 (1.7)
**Central Nervous System examination**			
Microcephaly	281 (43.6)	78 (56.9)	169 (36.6)
Developmental delay	180 (27.9)	48 (35.0)	118 (25.5)
Meningo-encephalitis	18 (2.8)	2 (1.5)	14 (3.0)

*on clinical examination

**based on any of the following: BERA/ASSR

### Rubella RT-PCR

OP swabs were collected from 105 of the 137 laboratory confirmed CRS cases. Of the 101 OP swabs tested, 44 (43.6%) were positive for rubella virus by RT-PCR. All the 20 representative strains selected for genotyping from different geographic regions, were clustered in 2B genotype (S2 Fig).

### Follow-up of laboratory confirmed CRS patients

We collected follow-up OP swabs from 45 laboratory confirmed CRS patients after a median of 11 (Interquartile range: 9–13) months of their diagnosis. Of these, eight patients were found RT-PCR positive indicating that these patients were still shedding the virus. These patients were aged between 11 and 16 months at the time of their follow-up sample collection.

### CRS surveillance quality indicators

Most of the suspected CRS patients (n = 586, 91%) were adequately investigated, while adequate specimens were collected from 87% of the patients. All specimens were transported to the laboratories within five days of collection. The results of serological investigations were reported within 4 days for 50% and within 10 days for 79.9% of sample collection. OP swabs were collected from 76.6% of the 137 laboratory confirmed CRS patients at the time of diagnoses. However, only 32.8% laboratory confirmed CRS patients could be followed-up for viral excretion. Only 54% laboratory confirmed CRS patients were detected within 3 months of birth (Table 3).

**Table 3:** Surveillance quality indicators.

Criteria	Threshold	%
**1. Reporting rate** (National annual rate of suspected CRS cases)	≥1/10 000	6.5–68.0 per 10,000 live births
**2. Adequacy of investigation**	>80%	90.9 (586/645)
(a) % suspected CRS with key data points		99.5 (642/645)
(b) % suspected CRS patients who underwent clinical evaluation for heart defects, eye signs and hearing.		91.2 (588/645)
**3. Specimen collection/testing adequacy** (% suspected cases with adequate specimen collected+ tested)	≥80%	87.0 (561/645)
**4. Adequacy of specimens for viral detection** (% confirmed cases with adequate specimens for virus detection)	≥80%	76.6 (105/137)
**5. Monitoring for virus excretion** (% confirmed CRS cases followed up for viral excretion)	≥80%	32.8 (45/137)
**6. Timeliness of detection** (% confirmed CRS cases detected within 3 months of birth)	≥80%	54.0 (74/137)
**7. Timeliness of specimen transport** (Proportion of specimens received at lab within 5 days of collection)	≥80%	100.0
**8. Timeliness of reporting laboratory results** (% test results reported within 4 days of receipt of specimen)	≥80%	50.1 (281/561)

## Discussion

During 2016–18, sentinel surveillance sites enrolled nearly 650 suspected CRS infants from five health facilities, about one fifth of whom were found to be laboratory confirmed, indicating the significance of rubella as a persistent public health problem in India. Occurrence of laboratory confirmed CRS patients indicates susceptibility of pregnant women to rubella infection and circulation of rubella virus in the community. CRS surveillance also met the quality indicators in terms of adequacy of investigation, adequacy of sample collection for serological diagnosis as well as virological confirmation. In long run, the data generated by this platform of sentinel surveillance could be useful to monitor the progress made towards control of rubella/CRS in India.

The majority of the suspected CRS patients in our surveillance were enrolled on account of the structural heart defect or eye signs, whereas only 13% were enrolled due to hearing impairment. Among the laboratory confirmed CRS patients, about 80% had heart structural defects while 60% had cataract, glaucoma and/or pigmentary retinopathy. Less than 40% of the laboratory confirmed CRS patients had hearing impairment. The low proportion of children with hearing impairment enrolled in our study could be due to failure to screen for hearing impairment in early infancy. In India, facilities for universal screening of newborns for hearing impairment before discharge as part of standard care are not available in most public hospitals. Among the five surveillance sites, only three were conducting routine universal screening of hearing. As a result, children with hearing impairment are detected late. Studies conducted in United Kingdom during 1978–82 have reported unacceptable delays in identifying hearing loss among newborn who were found to be IgM positive because of failure to perform auditory evoked response testing [13, 14]. Implementation of universal screening for hearing impairment at all health care facilities would significantly improve detection of suspected CRS cases with hearing impairment.

Our data indicated high mortality among patients with lab-confirmed CRS. The exact cause of death among these patients could not be determined. High mortality among CRS patients have been reported from South Africa (7–15%), and Vietnam (34%) [15, 16]. In Vietnam, mortality among CRS patients was associated with pulmonary hypertension, probably due to left-to-right shunting and CRS-induced vascular damage [16]. High mortality in CRS patients is an important issue that needs to be addressed in future surveillance in India.

The age distribution of mothers of CRS patients is an indication of susceptibility to rubella among women of child-bearing age. More than 88% of the mothers of the laboratory confirmed CRS patients detected in our surveillance were aged between 17 and 30 years. Mothers of 39 (28%) of the 137 laboratory confirmed CRS patients also had a history of febrile illness with rash during pregnancy. According to the serosurvey conducted among pregnant women from these five sentinel surveillance sites, 84.5% of mothers aged between 17 and 30 years were found to be sero-positive for rubella [17]. The measles-rubella vaccine campaigns targeting children aged between 9 months to 14 years are expected to increase the population immunity against rubella in India, thereby reducing rubella transmission and infection among susceptible pregnant women. Periodic serosurveys among pregnant women in sentinel sites could complement the CRS surveillance in providing data on susceptibility to rubella in women of reproductive age groups.

Our study had certain limitations. First, the variation in the reporting rates of suspected CRS across surveillance sites could be due to different sensitivity of surveillance system at each site. The proportion of suspected CRS patients enrolled on account of hearing impairment varied from 1.5% to 15% at different sites. We however did not estimate sensitivity of CRS surveillance across the sites. Second, the proportion of patients with hearing impairment was higher among lab-confirmed patients as compared to the discarded patients. This could be due to higher prevalence as well as due to higher proportion of lab confirmed CRS patients with suspected hearing impairment investigated for BERA/ASSR.

In conclusion, the findings of our sentinel surveillance indicate that CRS is an important public health problem in India. The CRS surveillance also met the targets for adequacy of investigations, sample collection and transportation to the laboratory. However, only half of the laboratory confirmed CRS cases were detected within 3 months of birth. This underscores the need for improving screening of newborns for congenital defects as well as hearing impairment. This will also improve the sensitivity of surveillance system. Till recently, India lacked a dedicated national surveillance system for birth defects [18]. Under the Rashtriya Bal Swasthya Karyakram, comprehensive newborn screening for eight visible birth defects has been initiated at the delivery points in many Indian States [19]. This platform for birth defect surveillance could be used for expanding CRS surveillance, by implementing universal screening for hearing impairment of newborns and screening for heart defects and eye signs.

## Supporting information

S1 Checklist STROBE checklist. (DOC)

S1 Fig Geographical distribution of laboratory confirmed CRS infants. (DOCX)

S2 Fig Phylogenetic tree. (DOCX)

S1 Table Description of sentinel sites. (DOCX)

S2 Table Characteristics of structural cardiac defects among laboratory confirmed CRS cases, 2016–18. (DOCX)
