# Supplementary figures and images for: Epidemiology of Congenital Rubella Syndrome (CRS) in India, 2016-18, based on data from sentinel surveillance
Source: PLoS Negl Trop Dis. 2020 Feb 3;14(2):e0007982. doi: 10.1371/journal.pntd.0007982 (PMC6996802; doi:10.1371/journal.pntd.0007982)

**S1 Fig: Geographical distribution of laboratory confirmed CRS infants**


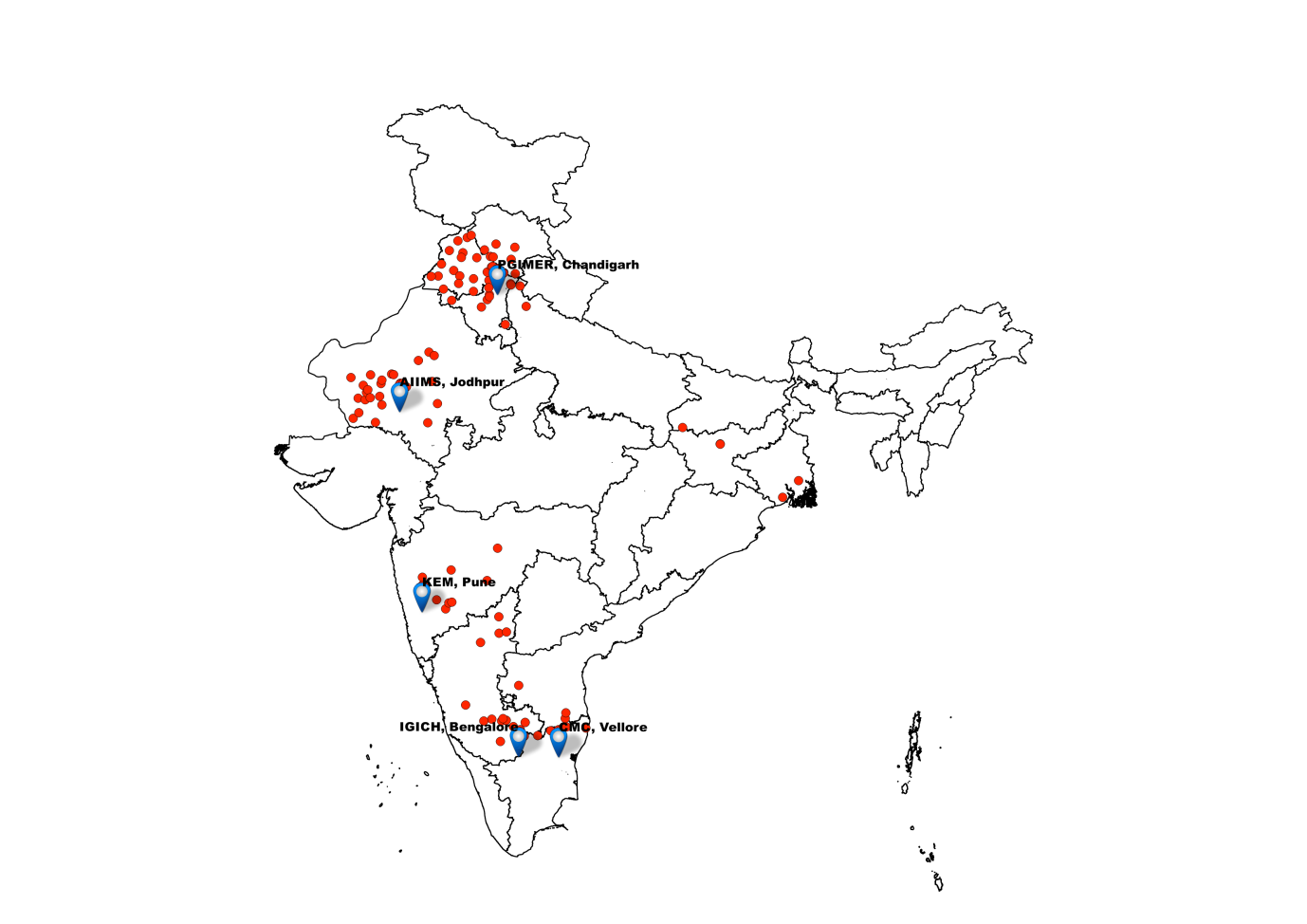

Supplement: S1 Fig — (DOCX) [file pntd.0007982.s002.docx]
