# Supplementary material for: Epidemiology of Congenital Rubella Syndrome (CRS) in India, 2016-18, based on data from sentinel surveillance
Source: PLoS Negl Trop Dis. 2020 Feb 3;14(2):e0007982. doi: 10.1371/journal.pntd.0007982 (PMC6996802; doi:10.1371/journal.pntd.0007982)
Supplement: S2 Fig — (DOCX) [file pntd.0007982.s003.docx]

**S2 Fig 2: Phylogenetic tree**
