# Supplementary material for: Epidemiology of Congenital Rubella Syndrome (CRS) in India, 2016-18, based on data from sentinel surveillance
Source: PLoS Negl Trop Dis. 2020 Feb 3;14(2):e0007982. doi: 10.1371/journal.pntd.0007982 (PMC6996802; doi:10.1371/journal.pntd.0007982)
Supplement: S1 Table — (DOCX) [file pntd.0007982.s004.docx]

**S1 Table: Description of sentinel sites**

| **Characteristics** | **AIIMS, Jodhpur** | **CMC, Vellore** | **KEM, Pune** | **IGICH, Bengaluru** | **PGIMER, Chandigarh** |
| --- | --- | --- | --- | --- | --- |
| Type of facility | Tertiary care hospital | Tertiary care hospital | Tertiary care hospital | Tertiary care children hospital | Tertiary care hospital |
| Location | Urban | Urban | Urban | Urban | Urban |
| Total OPD attendance (2018) | 3,86,764 | 22,46,664 | 1,36,663 | 1,48,876 | 27,25,183 |
| No. of births per year 2017 | 528 | 14469 | 2144 | 3205 | 5682 |
| No. of births per year 2018 | 1239 | 14140 | 2138 | 3300 | 5783 |
| No. of beds | 684 | 2282 | 550 | 475 | 1850 |
