# Supplementary material for: Epidemiology of Congenital Rubella Syndrome (CRS) in India, 2016-18, based on data from sentinel surveillance
Source: PLoS Negl Trop Dis. 2020 Feb 3;14(2):e0007982. doi: 10.1371/journal.pntd.0007982 (PMC6996802; doi:10.1371/journal.pntd.0007982)
Supplement: S2 Table — (DOCX) [file pntd.0007982.s005.docx]

**S2 Table: Characteristics of structural cardiac defects among laboratory confirmed congenital rubella syndrome cases — Congenital Rubella Sentinel Surveillance, India, November 2016– December 2018**

| **Type of defects*** | **Laboratory-confirmed CRS with structural heart defects (n = 108)** |
| --- | --- |
| **Single cardiac defects** |  |
| PDA | 44 (63.8) |
| ASD/PFO | 12 (17.4) |
| VSD | 12 (17.4) |
| PS | 1 (1.5) |
| **Total** | **69 (63.9)** |
| **Complex cardiac defects** |  |
| Complex defects with PDA | 30 (76.9) |
| Complex defects with PS, without PDA | 2 (5.1) |
| Tetralogy of Fallot | 1 (2.6) |
| Complex defects with ASD/VSD, without PDA / PS | 6 (15.4) |
| **Total** | **39 (36.1)** |

Abbreviation: CRS = congenital rubella syndrome; PDA = patent ductus arteriosus; ASD = atrial septal defect; PFO = patent foramen ovale; VSD = ventricular septal defect; PS = pulmonary stenosis;

Classified as simple or complex defects according to the NIH National Heart, Lung, and Blood Institute definition of Types of Congenital Heart Defects. Available at: <https://www.nhlbi.nih.gov/health-topics/congenital-heart-defects>
